# Retinal pigment epithelial cells reduce vascular leak and proliferation in retinal neovessels

**DOI:** 10.1007/s10456-024-09954-4

**Published:** 2024-11-27

**Authors:** Simone Tzaridis, Edith Aguilar, Michael I. Dorrell, Martin Friedlander, Kevin T. Eade

**Affiliations:** 1 The Lowy Medical Research Institute, La Jolla, CA USA; 2 Department of Molecular Medicine, The Scripps Research Institute, La Jolla, CA USA; 3 Point Loma Nazarene University, San Diego, CA USA

**Keywords:** Epithelial-mesenchymal transition, Neovascularization, Macular telangiectasia type 2, Pigment migration, Vascular leakage, Retinal imaging

## Abstract

In multiple neurodegenerative diseases, including age-related macular degeneration, retinitis pigmentosa, and macular telangiectasia type 2 (MacTel), retinal pigment epithelial (RPE)-cells proliferate and migrate into the neuroretina, forming intraretinal pigment plaques. Though these pigmentary changes are hallmarks of disease progression, it is unknown if their presence is protective or detrimental.

Here, we first evaluated the impact of pigment plaques on vascular changes and disease progression in MacTel. In a retrospective, longitudinal study, we analyzed multimodal retinal images of patients with MacTel and showed that pigment plaques were associated with decreased vascular leakage and stabilized neovascular growth. We then modeled the underlying pathomechanisms of pigment plaque formation in aberrant neovascular growth using the very-low-density lipoprotein receptor mutant (*Vldlr*^−/−^) mouse. Our data indicated that during RPE-proliferation, migration and accumulation along neovessels RPE-cells underwent epithelial-mesenchymal transition (EMT). Pharmacologic inhibition of EMT in *Vldlr*^−/−^ mice decreased pigment coverage, and exacerbated neovascular growth and vascular leakage.

Our findings indicate that the proliferation, migration and perivascular accumulation of RPE-cells stabilize vascular proliferation and exudation, thereby exerting a protective effect on the diseased retina. We conclude that interfering with this “natural repair mechanism” may have detrimental effects on the course of the disease and should thus be avoided.

**Supplementary Information:**

The online version contains supplementary material available at 10.1007/s10456-024-09954-4.

## Introduction

Retinal pigment epithelial (RPE)-cells possess numerous functions, including response to stress and damage of the neuroretina [1]. In many neurodegenerative diseases, including age-related macular degeneration (AMD), retinitis pigmentosa, and macular telangiectasia type 2 (MacTel), RPE-cells have been shown to proliferate and migrate into the neuroretina, forming intraretinal pigment plaques [2, 3]. Increasing evidence suggests that during this process, RPE-cells transition from an epithelial to a mesenchymal state (“epithelial-mesenchymal transition”, EMT), granting these cells mesenchymal properties such as the ability to proliferate and migrate [4, 5]. Though pigmentary changes are commonly associated with disease progression, their role is not fully understood. This association could represent a causative relationship whereby the pigmentary changes contribute to disease progression. Alternatively, pigmentary changes could be protective from worsening disease, or pigmentation could simply be an effect of other disease-causing retinal changes that are unrelated to disease progression.

MacTel is an example of a progressive, neurodegenerative retinal disease that affects the central retina. Secondary vascular alterations are commonly observed [6]. Pigment plaques are a frequent finding in MacTel and pigmented changes and vascular alterations have been found to co-localize [2, 7]. Early vascular changes of the disease include telangiectasia and increased vascular leakage, indicating a dysfunctional inner blood-retina barrier [6]. With disease progression, a shift of vessels from the deep retinal plexus to the outer retina as well as the formation of retinal-choroidal anastomoses and outer retinal neovascularization has been described. These changes may precede the infrequently observed formation of vision-threatening exudative subretinal neovascularization [8–11]. The origin of neovessels has been ascribed to the retinal, rather than the choroidal, vasculature [12–15]. Recent imaging studies in MacTel proposed that the formation of outer retinal neovascularization induced proliferation of the RPE, once the RPE and outer retinal vessels come in contact with one another [16]. It has been proposed that RPE cells then use these abnormal vessels as a scaffolding to migrate into inner retinal layers, where they form dense pigmented plaques [7]. Their role during disease progression and their impact on vascular changes, including vascular leakage and proliferation, have not yet been evaluated. We hypothesized that the perivascular accumulation of RPE-cells may stabilize vascular proliferation and reduce vascular permeability.

The very-low-density lipoprotein receptor (*Vldlr*) mutant (*Vldlr*^–/–^) mouse is a rodent model of subretinal neovascularization and is used to study disease characteristics of MacTel, retinal angiomatous proliferation and other conditions [17, 18]. As in MacTel, in *Vldlr*^−/−^ mice, neovascular changes originate from the retinal vasculature. Retinal vessels proliferate, grow to the outer retina, and subsequently form subretinal neovascularization. As the disease progresses, mice show a proliferation of RPE cells with accumulation along neovascular tufts, followed by the migration of pigmented cells along retinal vessels into the neuroretina [3, 17, 19]. These events mirror the key neovascular and pigmentation-related events observed in MacTel and other diseases with subretinal neovascularization, making the *Vldlr*^−/−^ mouse an excellent model to study these changes.

In this study, we aimed to (I) evaluate the role of pigment plaques on vascular changes and disease progression in MacTel, and (II) study underlying disease mechanisms using the *Vldlr*^−/−^ mouse model mirroring these changes.

First, we show in a longitudinal, retrospective study of eyes with MacTel that perivascular pigment accumulation was associated with reduced vascular leakage and decreased de novo formation of exudative neovascularization. We then explored whether the observed associations were causally related and which mechanisms led to perivascular pigment accumulation in *Vldlr*^−/−^ retinas. We found an enrichment of EMT-inducers and key mesenchymal markers in the RPE of *Vldlr*^−/−^ mice. Pharmacologic inhibition of EMT-inducers led to decreased perivascular pigment accumulation and enhanced neovascular growth and exudation in the *Vldlr*^−/−^ model, indicating a protective effect of pigmentary changes on vascular proliferation, mitigating vascular leak and proliferation. Based on our findings, we propose that EMT of the RPE, followed by proliferation, migration and perivascular accumulation may function as a “natural repair mechanism”, exerting beneficial, anti-angiogenic and anti-exudative effects on the diseased retina. As such, we conclude that interfering with these mechanisms may have detrimental effects on the course of the disease and should, thus, be avoided.

## Results

### Perivascular accumulation of pigment is associated with decreased vascular leakage in eyes with MacTel

To investigate the impact of perivascular pigment accumulation on vessel leakage and proliferation in MacTel, we compared the longitudinal courses of eyes with and eyes without pigment plaque de novo formation. A total of 1216 eyes from 608 patients of 12 study centers were evaluated. 69 eyes from 69 patients (mean age 61.9 [range 53–71] years; 37 [54%] females) were included and reviewed over a mean period of 41.6 months (range 24–60). 35 eyes (51%) showed a de novo development of pigmentary changes, and 34 eyes did not. Pigment plaques predominantly accumulated along vessels within the temporal parafovea (ETDRS subfield 5), usually sparing the fovea (Online Resource 1). Rarely, an extension of changes to the superior, inferior, or nasal parafovea (ETDRS subfields 2, 4, 3) was observed.

**Fig. 1:**
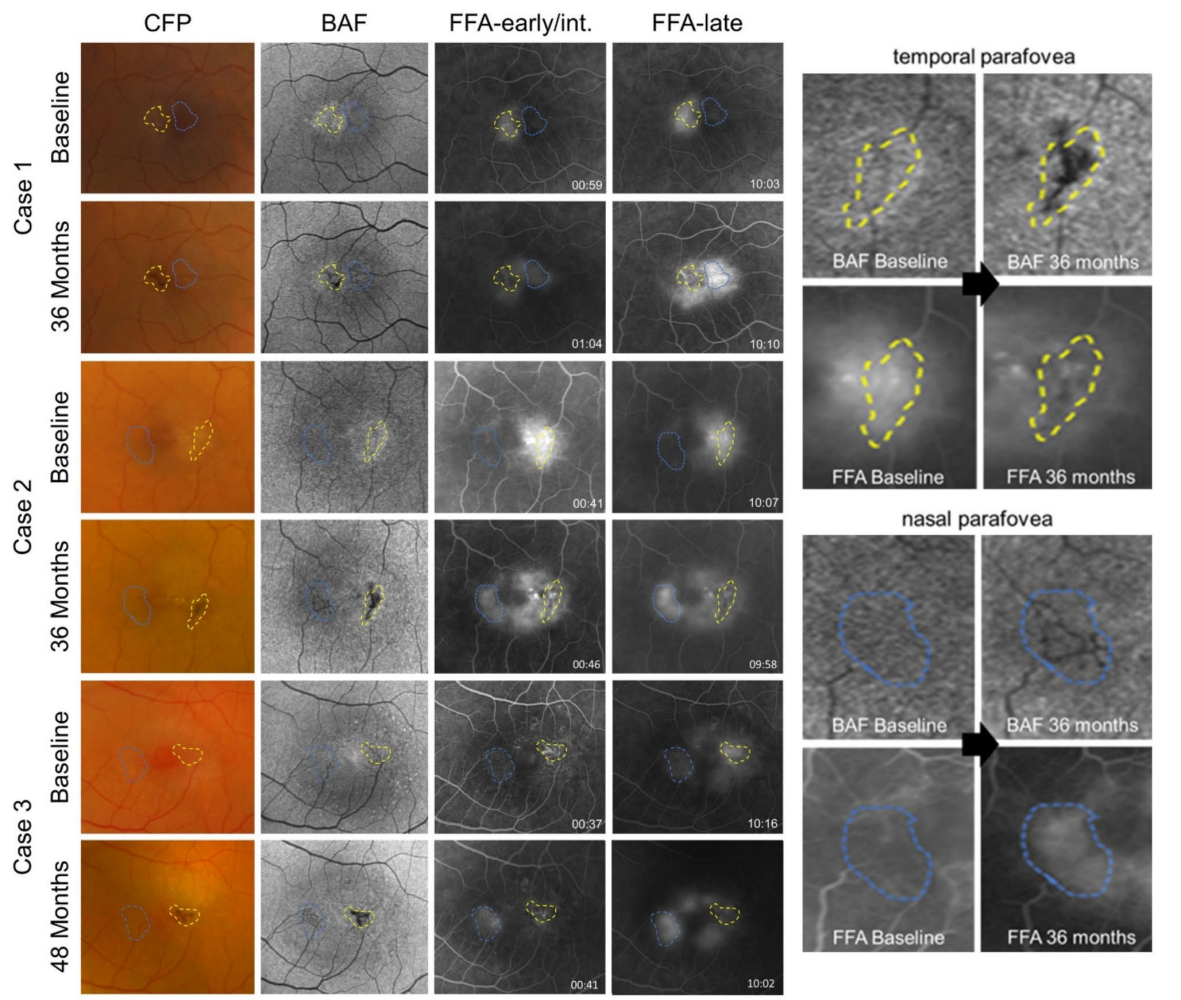
Perivascular accumulation of pigment plaques decreases vascular leakage in patients with MacTel. Longitudinal courses of three exemplary eyes showing a de novo formation of perivascular pigment plaques, imaged with color fundus photography (CFP), blue-light autofluorescence (BAF), and fundus fluorescein angiography (FFA; early to intermediate phase and late phase). A decrease in fluorescein leakage can be observed in vessels covered with pigment (yellow borderline), while vessels lacking pigment may show an increase in leakage and proliferation (blue borderline). The right column illustrates enlarged BAF and FFA images of case #2 within the temporal and nasal parafovea, respectively

**Fig. 2:**
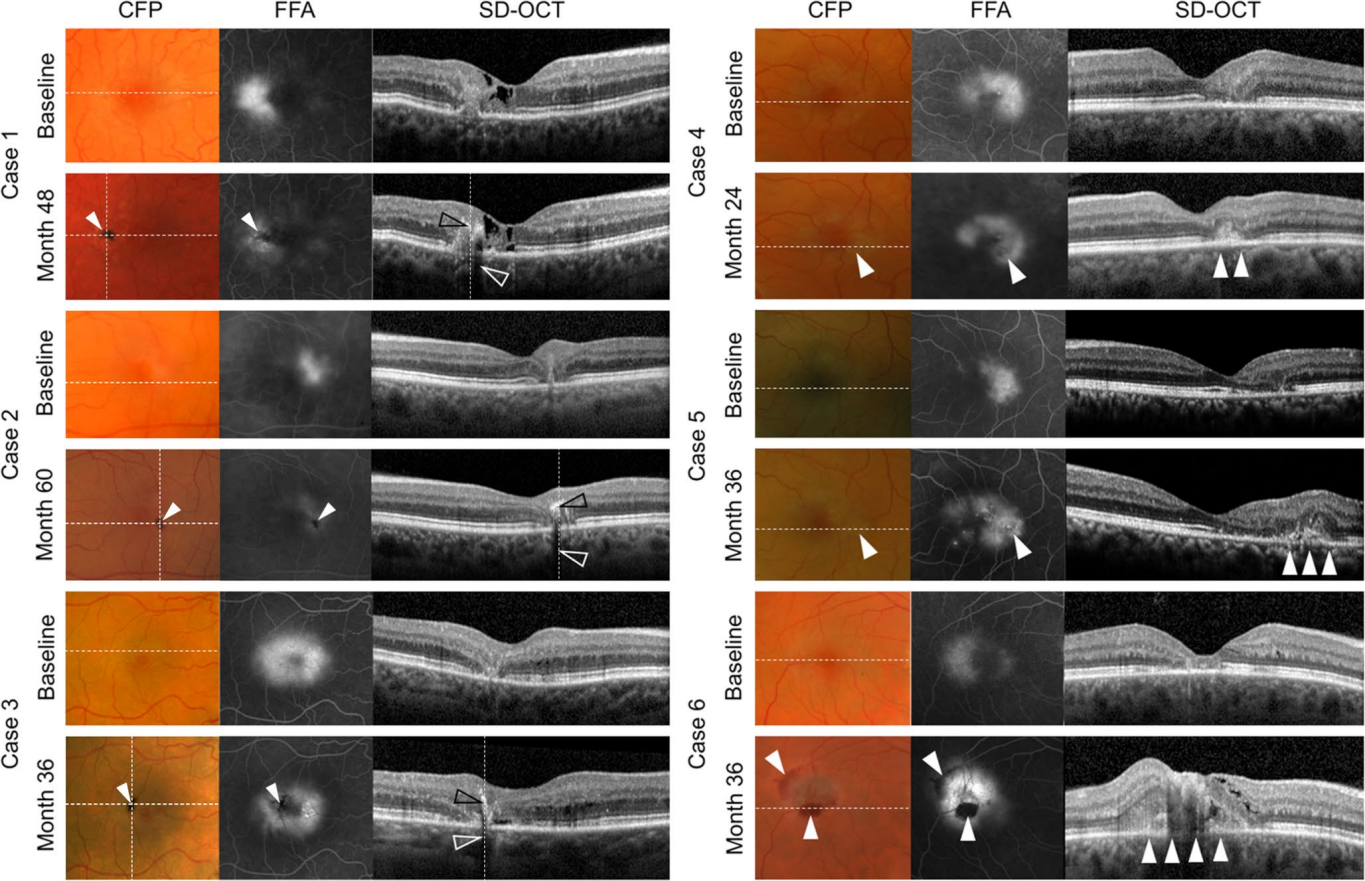
Perivascular pigment accumulation stabilizes vessel growth and decreases neovascular exudation in eyes with MacTel. Longitudinal courses of exemplary eyes with (cases 1–3) and without (cases 4–6) a de novo development of pigment plaques. Perivascular pigment accumulation is associated with a decrease in fluorescein leakage on fundus fluorescein angiography (FFA), and overall stable findings on spectral domain-optical coherence tomography (SD-OCT). Note the increase in intraretinal hyper-reflectivity (black arrowheads) and shadowing of underlying structures (white arrowheads, OCT-scans, left panel) associated with the de novo development of pigment plaques (solid white arrowheads, CFP and FFA images, left panel). Eyes without pigmentary changes show an increase in fluorescein leakage that may be associated with the de novo development of exudative neovascularization (solid white arrowheads, right panel). Hemorrhages may occur. White dashed horizontal lines indicate the position of OCT-B-scans on corresponding CFP images; vertical lines indicate the position of pigment plaques on CFP and OCT-scans, respectively. CFP: color fundus photography

Longitudinal courses of eyes developing pigmented lesions differed from those without. A decrease in fluorescein leakage and stabilization of vessel proliferation was noted in all but one eye with pigment plaques. The observed effects were, however, focal and limited to vessels covered with pigment. In these eyes, vessels lacking pigment plaques showed stable, or rarely, increased leakage (observed in the nasal parafovea (ETDRS subfield 3) of 4/35 eyes; see Fig. 1). Notably, coverage of vessels with pigment was associated with a decrease in fluorescein leakage both in the early and late phase of fundus fluorescein angiography (FFA), suggesting a sealing, rather than a mere shadowing effect associated with perivascular pigment accumulation. In eyes without pigmentary changes, an increase in vascular leakage (in 16/34 eyes [47%]) or stable leakage (in 18/34 eyes [53%]; Table 1) was observed. Proliferation of vessels and increase in leakage were primarily observed within the temporal parafovea (ETDRS subfield 5). On optical coherence tomography (OCT), the de novo development of pigment plaques (see Fig. 2, left panel, cases 1–3) was associated with overall stable findings. Compared to the baseline visit, an increase in intraretinal hyper-reflectivity as well as increased shadowing of underlying structures within areas showing pigment plaques formation could, however, commonly be observed. Whether the latter signals on OCT were associated with perivascular pigment accumulation, vascular changes or a combination of both events can, however, not be determined, as discussed in previous imaging studies [16, 20, 21]. Exemplary longitudinal courses are illustrated in Figs. 1 and 2.

Exudative subretinal neovascularization is considered a severe, vision-threatening complication of MacTel, and is associated with severe vascular leakage. The de novo formation of exudative subretinal neovascular membranes was less frequently observed in eyes with, compared to eyes without, pigment plaques (in 1/35 [3%] eyes vs. 7/34 [21%]; Fisher’s exact test, *p* = 0.0275). Table 1 gives an overview of the clinical findings in this study cohort.

**Table 1:** Clinical findings in the study cohort of patients with macular telangiectasia type 2 (MacTel)

	Pigment plaques (no. [%])	No pigment plaques (no. [%])	Statistical significance
Eyes	35 [51%]	34 [49%]	Ns^a^
Follow up period (months; mean [range])	41 [24–60]	39 [24–60]	Ns^a^
Fluorescein leakage temporal parafovea (ETDRS subfield 5)			
Increase	1 [3%]	16 [47%]	*p* < 0.0001^b^
Decrease	34 [97%]	0	NA
Stable	0	18 [53%]	NA
Exudative neovascularization	1 [3%]	7 [21%]	*p* = 0.0275^b^
Visual acuity: Loss of letters/year (mean [SD])	1.1 [2.3]	3.1 [3.9]	*p* = 0.0125^a^

^a^ p-values were calculated based on an unpaired t-test with Welch’s correction

^b^ p-values were calculated based on a Fisher’s exact test. Ns: not significant; NA: not applicable

In summary, patients with MacTel showed (I) an accumulation of pigment plaques along abnormal retinal and subretinal vessels, (II) a decrease in vascular leakage that was associated with the development of pigment plaques, and (III) a decrease in exudative subretinal neovascularization associated with the presence of pigment plaques. Based on our findings in patients with MacTel, we hypothesized that: (I) Proliferating vessels may trigger the proliferation and perivascular accumulation of pigment; (II) Pigment plaques may be formed by RPE cells that undergo EMT, proliferate, migrate and accumulate along proliferating vessels; and (III) Perivascular pigment plaques may decrease vascular leakage and stabilize vessel proliferation, thus having a beneficial effect on the diseased retina.

To test these hypotheses, and to further evaluate disease mechanisms leading to perivascular pigment accumulation, we studied related changes in the *Vldlr*^−/−^ mouse model. Similar to eyes with MacTel, the *Vldlr*^−/−^ mouse model shows a proliferation of retinal vessels, formation of retinal-choroidal anastomoses and subretinal neovascularization. With disease progression, RPE-cells proliferate and accumulate along subretinal neovessels, and subsequently migrate along retinal vessels into the neuroretina [17–19]. 

### Proliferating retinal vessels trigger perivascular pigment accumulation

**Fig. 3:**
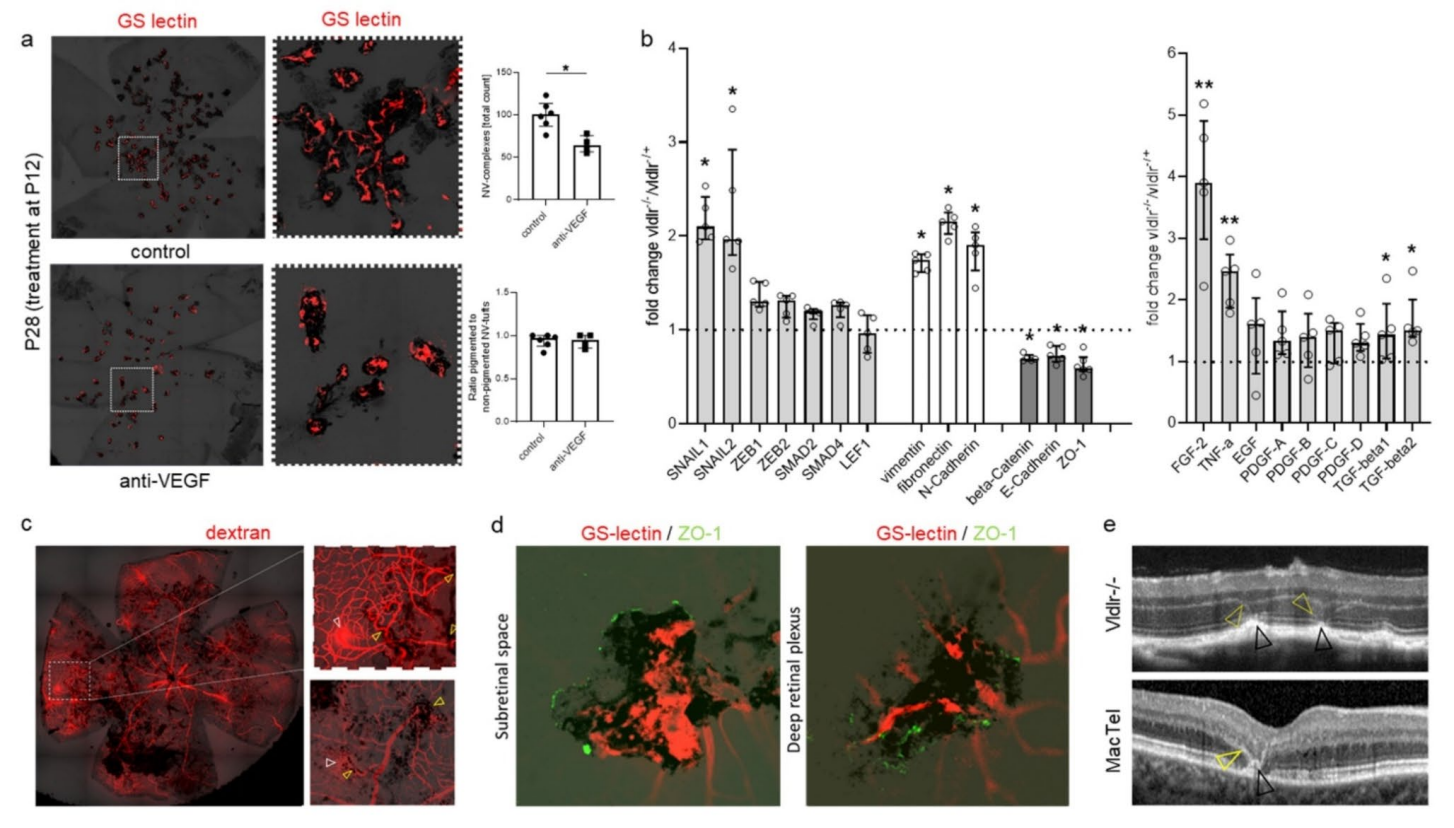
The Vldlr knockout mouse model mirrors vascular and pigmentary changes observed in MacTel. **a**: Proliferating vessels trigger perivascular pigment accumulation in *Vldlr*^−/−^ mice. Neovascular (NV) tufts and perivascular pigment accumulation were analyzed using GS-lectin staining and bright field images in P28 *Vldlr*^−/−^ mice treated with intravitreal injections of anti-VEGF (*n* = 6) or control IgG (*n* = 4) at P12. While anti-VEGF treatment significantly decreased the total number of NV-tufts (Mann-Whitney test, *p = 0.019), the ratio of pigmented to non-pigmented NV-tufts was unchanged. Pigment plaques only developed along proliferating vessels. Error bars indicate the median and interquartile range. **b**: Regulation of different genes coding for key molecules and inducers of epithelial-mesenchymal transition (EMT) in the retina and RPE of *Vldlr*^−/−^ mice at P42. Changes in genes between *Vldlr*^–/–^ and *Vldlr*^−/+^ mice, as analyzed using a PCR array for EMT, are shown. P values were calculated based on a Mann-Whitney test of the replicate 2(-Delta Ct) values for each gene in the *Vldlr*^−/−^ and Vldlr^−/+^ groups. *P < 0.05, **P < 0.01 (*n* = 5 each). Median and IQR are shown for each gene. **c**: Dextran-angiography in a 12-months-old *Vldlr*^−/−^ mouse depicts vessel and pigment proliferation and migration of pigmented cells along retinal vessels. Vessels covered with pigment show reduced dextran leakage (enlarged images, yellow arrowheads) compared to vessels not covered with pigment (white arrowheads). **d**: Pigment plaques cover proliferating vessels (stained with GS-lectin) and express ZO-1, indicating the formation of tight junctions along subretinal and retinal vessels in eyes of 10-months-old *Vldlr*^−/−^ mice. **e**: *Vldlr* knockout (*Vldlr*^−/−^) mice show hyper-reflective changes at the level of the outer retina / retinal pigment epithelium (RPE; yellow and black arrowheads, respectively) on optical coherence tomography that resemble alterations observed in MacTel

We first set out to investigate whether vascular proliferation triggers the proliferation and perivascular accumulation of pigment. In the *Vldlr*^−/−^ mouse model, retinal vessels begin proliferating around P12, followed by the growth of retinal vessels to the outer retina, and the formation of subretinal neovascular complexes around P16-P21 [22]. RPE-cells start proliferating around 4 weeks of age, accumulate along neovessels in the subretinal space, and subsequently migrate along retinal vessels into the neuroretina [18, 19, 23]. By inhibiting vascular proliferation using neutralizing antibodies against vascular endothelial growth factor (VEGF), we found a reduction in neovascular tuft formation. The ratio of pigmented to non-pigmented neovessels was, however, unchanged, and pigment plaques only developed along proliferating neovessels (see Fig. 3a), suggesting that neovessels precede, and are required for, pigment plaque formation.

### Pigment plaques are formed by RPE cells in Vldlr−/− retinas and express similar markers as observed in eyes with MacTel

Previous findings in postmortem retinal samples of eyes with MacTel or retinitis pigmentosa indicated that intraretinal pigment plaques originated from the RPE [2]. Intraretinal lesions were found to express the epithelial cell marker cytokeratin18 (CK18), that is specific to RPE-cells in the retina, but were negative for RPE65. Markers for mesenchymal cells (alpha-smooth muscle actin [ASMA]) and macrophages/ microglia (IBA1) were also evaluated, but found to be absent [2]. To verify these findings in the *Vldlr*^−/−^ mouse model, we evaluated similar markers as previously described [2]. Proliferating RPE cells within the subretinal space expressed CK18 and RPE65. Some, but not all of these cells also showed immunoreactivity for ASMA (see Online Resource 2), indicating EMT of the RPE. Intraretinal pigment plaques, on the other hand, expressed CK18, but neither RPE65 nor IBA1 were detected (see Online Resources 2 and 3). The expression of ASMA was observed in single intraretinal pigmented lesions. The latter were, however, overall smaller and less dense compared to lesions lacking ASMA expression, indicating a transitional, possibly less mature, stage of these lesions (Online Resources 2 and 3).

### RPE-cells undergo EMT in Vldlr−/− retinas

Under physiologic conditions, the RPE is formed by a monolayer of polarized cells. Disintegration of the RPE monolayer and proliferation and migration of RPE-cells have been described in several degenerative retinal diseases, and have been attributed to RPE cells transitioning from an epithelial to a mesenchymal state [5]. To test whether RPE cells underwent EMT in the *Vldlr*^−/−^ mouse model, we compared gene expression levels of known EMT-related genes in the RPE of *Vldlr*^–/–^ mice and control *Vldlr*^−/+^ littermates at P42. At this timepoint, RPE cells have been shown to proliferate and accumulate along subretinal neovessels and start migrating along retinal vessels into the neuroretina [17–19]. Using qPCR arrays, we found an enrichment of genes coding for EMT pathways (SNAIL1/2) and different mesenchymal markers (vimentin, fibronectin, N-cadherin) in the RPE of *Vldlr*-/- mice. Genes coding for epithelial markers (beta-catenin, E-cadherin, zonula occludens-1 [ZO-1]), on the other hand, were decreased (Fig. 3b), indicating that RPE cells underwent EMT in this model.

Next, we tested mRNA expression levels of known inducers of EMT in the retinas and RPE of *Vldlr*^−/−^ mice. The largest differences between *Vldlr*^–/–^ and heterozygous control littermates were found in fibroblast growth factor-2 (FGF2), which was increased 4-fold, and in tumor-necrosis factor-alpha (TNFA), which was increased 2.5-fold. FGF2 is a known driver of EMT that, among other factors, has been described to play a role in inducing EMT in RPE cells in proliferative vitreoretinopathy (PVR) [24]. FGF2 is also known to play a role in inducing subretinal fibrosis, and has been shown to have pro-angiogenic properties [25, 26]. TNFA is a proinflammatory cytokine and a known inducer of EMT in RPE cells [5, 27]. Elevated levels of TNFA have been detected in vitreous samples and epiretinal membranes of patients with PVR [27–29]. In vitro, TNFA has been shown to induce RPE cells to upregulate EMT markers and mesenchymal key molecules [30]. Increased expression levels of TNF have previously been found in the retinas of *Vldlr*^−/−^ mice, and in particular, at the level of the deep retinal plexus [22]. 

### Perivascular pigment decreases neovascular leakage and proliferation in Vldlr−/− retinas

**Fig. 4:**
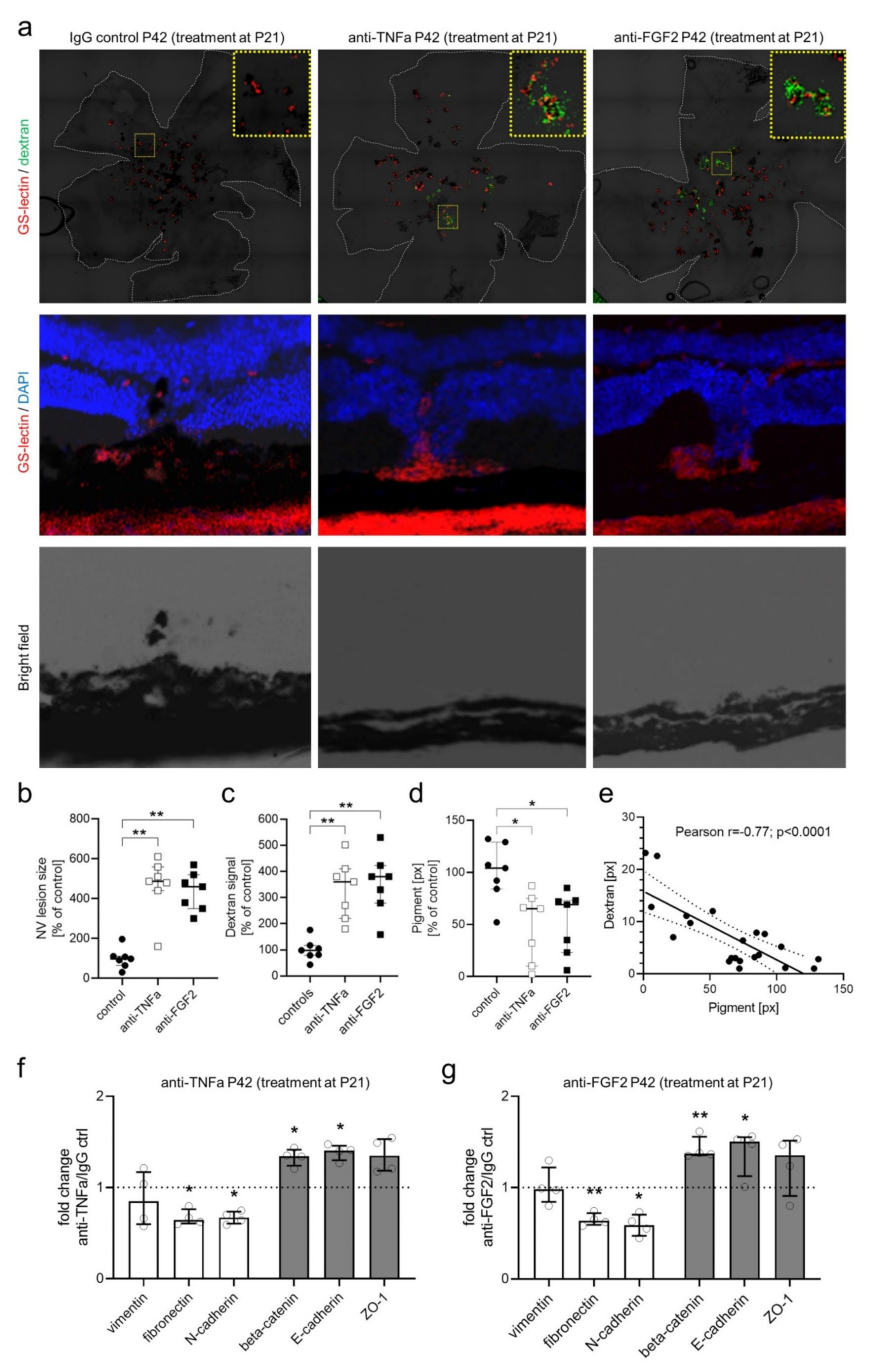
Inhibition of epithelial-mesenchymal-transition (EMT) leads to enhanced neovascular proliferation and leak in the *Vldlr*^−/−^ model. **a**-**c**: Dextran angiography and GS lectin staining in flat mounted (a, upper panel) or cryo-sectioned (a, middle and lower panel) *Vldlr*^−/−^ mice treated with intravitreal injections of neutralizing antibodies against TNFA (*n* = 7) or FGF2 (*n* = 7) at P21 showed a significant increase in dextran leakage (a, upper panel; c), a significant increase in size of neovascular (NV) complexes (b), and a significant decrease in pigment accumulation (number of pixels positive for pigment; d) at P42 compared to IgG-treated controls (*n* = 7). Yellow-dotted boxes in a (upper panel) show enlarged neovascular complexes in treated eyesGS-lectin staining and bright field images of cryo-sectioned Vldlr−/− (a, middle and lower panel) illustrate decreased pigment proliferation and perivascular accumulation in eyes treated with anti-TNFA or anti-FGF2. e: Dextran-leakage (number of pixels positive for dextran) was negatively correlated (Pearson r = − 0.78; p < 0.0001) with pigment accumulation (number of pixels positive for pigment) in flat-mounted Vldlr−/− eyes. f and g: Changes in genes coding for key molecules of EMT in Vldlr–/– mice at P42 treated with either anti-TNFA (f) or anti-FGF2 (g) compared to IgG-treated Vldlr−/− mice (treatment at P21), as analyzed using a PCR array for EMT, are shown. While mesenchymal key molecules (white bars) were downregulated, epithelial key molecules were enriched (grey bars). P values were calculated based on a Mann-Whitney test of the replicate 2(-Delta Ct) values for each gene. *P < 0.05, **P < 0.01 (n = 4 each). GS-lectin staining and bright field images of cryo-sectioned *Vldlr*^−/−^ (a, middle and lower panel) illustrate decreased pigment proliferation and perivascular accumulation in eyes treated with anti-TNFA or anti-FGF2. e: Dextran-leakage (number of pixels positive for dextran) was negatively correlated (Pearson *r*=-0.78; *p* < 0.0001) with pigment accumulation (number of pixels positive for pigment) in flat-mounted *Vldlr*−/−eyes. f and g: Changes in genes coding for key molecules of EMT in *Vldlr*–/–mice at P42 treated with either anti-TNFA (f) or anti-FGF2 (g)compared to IgG-treated *Vldlr*^−/−^ mice (treatment at P21), as analyzed using a PCR array for EMT, are shown. While mesenchymal key molecules (white bars) were downregulated, epithelial key molecules were enriched (grey bars). P values were calculated based on a Mann-Whitney test of the replicate 2(-Delta Ct) values for each gene. *P < 0.05, **P< 0.01 (n= 4 each)

Similar to eyes with MacTel, we found that in the *Vldlr*^−/−^ mouse model vessels covered with pigment showed reduced dextran leakage compared to vessels not covered with pigment (Fig. 3c). Perivascular pigment plaques expressed zonula occludens-1 (ZO-1), indicating the formation of tight junctions around proliferating vessels, thereby possibly reducing vascular leakage (Fig. 3d). Furthermore, on OCT, *Vldlr*^−/−^ mice showed hyper-reflective changes at the level of the outer retina/ RPE that resemble alterations observed in MacTel (Fig. 3e). These changes have been proposed to represent outer retinal neovascularization and proliferating RPE-cells [16]. Next, we set out to investigate whether inhibiting EMT of the RPE may impact neovascular leakage and proliferation in the *Vldlr*^–/–^ model. Mice treated with neutralizing antibodies against FGF2 or TNFA from P21 to P42 showed a significant increase in vascular leakage and in the size of neovascular complexes as well as a significant decrease in perivascular pigment accumulation at P42 in comparison to IgG-treated control animals (Fig. 4a-d). Vascular leakage and perivascular pigment accumulation showed a negative correlation in both treated and control animals (Fig. 4e).

To test whether the observed morphological changes were associated with the inhibition of EMT we compared gene expression levels of EMT-related genes in the RPE of *Vldlr*^−/–^ mice treated with FGF2, TNFA or control IgG. While no changes were observed for EMT pathways, genes coding for epithelial markers were enriched, and mesenchymal markers were decreased in animals treated with FGF2 or TNFA, indicating the inhibition of EMT (Fig. 4f-g).

**Fig. 5:**
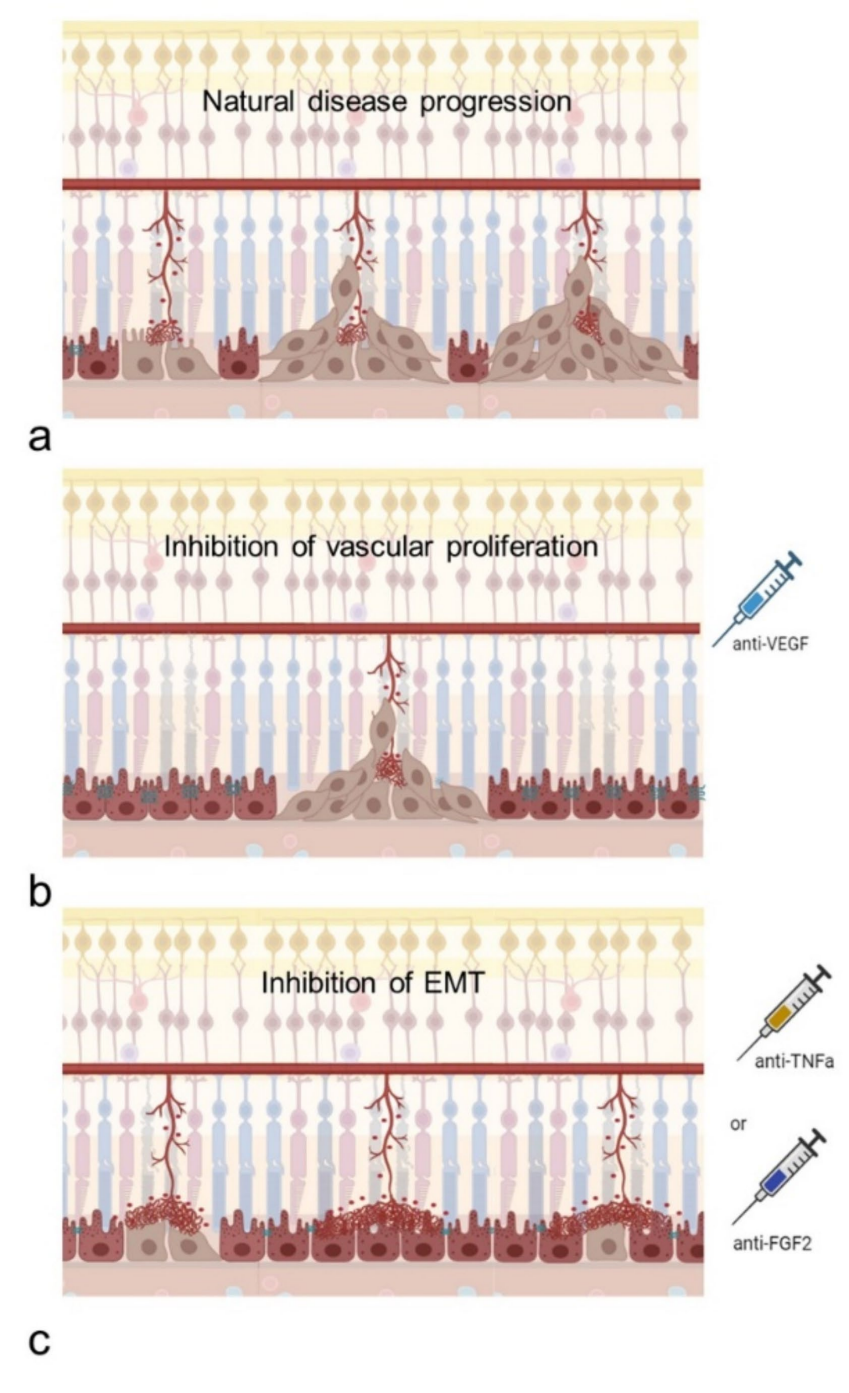
Schematic illustration of the proposed interplay between RPE-cells and retinal vessels in the *Vldlr*^−/−^ model. **a**: Proliferating vessels of the deep retinal plexus grow to the outer retina, form neovascular tufts and come in contact with the RPE. RPE cells transition from an epithelial to a mesenchymal state, proliferate and migrate along retinal vessels into the neuroretina, forming perivascular plaques. **b**: Eyes treated with intravitreal antibodies against VEGF (anti-VEGF) show a reduction in vessel proliferation and decreased numbers of neovascular tufts, while EMT of the RPE is not impacted. **c**: In eyes treated with intravitreal antibodies against TNFa or FGF2, epithelial-mesenchymal transition (EMT) of the RPE is inhibited. RPE cells show reduced proliferation, perivascular accumulation and migration. Neovascular tufts show more leakage and increased lateral growth. Created with BioRender.com

In summary, we suggest that the perivascular accumulation of RPE-cells may stabilize neovascular proliferation and leakage, thereby exerting a beneficial, protective effect on the diseased retina. Figure 5 summarizes the herein proposed mechanisms in a schematic illustration.

## Discussion

Pigment plaques are associated with a variety of neurovascular degenerative diseases of the retina. The formation of these plaques has been attributed to RPE-cells that accumulate along subretinal and retinal vessels, migrating into the neuroretina [2, 3]. The role these changes may play during disease progression is, however, not yet understood. In this study, we sought to evaluate the impact of perivascular pigment plaques on the progression of vascular changes in eyes with MacTel. We observed an association of perivascular pigment with a focal decrease in vascular permeability, and stabilization of vascular proliferation. The development of exudative subretinal neovascular complexes was observed less frequently in eyes with, compared to eyes without, pigment plaque formation. There is no clear agreement in the literature about the extent of these potential associations between pigment plaques and subretinal neovascularization in MacTel. Leung et al. found that in about 25% of neovascular eyes, subretinal neovascularization coincided with or preceded pigmentary changes [7]; Engelbrecht et al. observed that pigmented lesions preceded the development of neovascular membranes in 100% of their cases (in 11/11 eyes) [13]. Meleth et al. reported the de novo-development of neovascularization in equal numbers of eyes with and without pre-existing pigmentations [31]. Differences between our data and previous reports may be explained by differing definitions of “neovascularization” and “exudation” based on distinct imaging modalities. Different inclusion and exclusion criteria can also produce distinct study populations with varying disease stages, baseline characteristics and observational periods. More recent studies evaluating longitudinal OCT-A data found that the formation of outer retinal neovascularization preceded perivascular pigment accumulation in all cases [20]. Based on their findings on multimodal retinal imaging, Mueller et al. suggested that outer retinal neovascularization induced proliferation of the RPE, once retinal vessels came in contact with the RPE [16], a finding that is similar to previous observations in *Vldlr*^−/−^ mice and other models [17–19, 32]. Our results are in line with these later findings of neovascularization preceding pigmentation and indicate that perivascular pigment accumulation is a focal process, possibly triggered by vascular leakage and proliferation, and may represent a “repair mechanism” of the disrupted RPE. Furthermore, our results may indicate that pigment plaques could be used as a biomarker to predict neovascular exudation and vascular stabilization, respectively. Prospective, longitudinal studies are needed to verify our findings.

The reason why only a subset of MacTel patients develop pigment plaques currently remains obscure and warrants further studies. Limited observational periods and patient numbers might have confounded our findings, representing potential limitations of this study.

To study underlying disease mechanisms, we evaluated related pigmentary and vascular changes in the *Vldlr*^−/−^ mouse model. We showed that perivascular pigment plaques expressed similar markers as previously described in postmortem retinal specimens of eyes with MacTel and other diseases, verifying that these changes are formed by RPE-cells [2, 3]. We confirmed that pigment accumulation was associated with a decrease in vessel leakage in the *Vldlr*^−/−^ model. Our findings indicate that perivascular pigment plaques may form tight junctions around proliferating vessels, as indicated by ZO-1 localization, thereby reducing vascular leakage and stabilizing neovascular growth. A similar effect of pigment plaques on vessel permeability has been previously reported by Jaissle et al., where migrating RPE-cells were observed to form tight junctions with vascular endothelial cells, thus sealing retinal vessels in a mouse model for retinitis pigmentosa [32]. 

While dense pigment plaques within the neuroretina and subretinal space of *Vldlr*^−/−^ mice showed a focal expression of ZO-1, genes coding for the same epithelial cell marker were downregulated in the RPE of *Vldlr*^−/−^ mice, indicating a broader loss of cell-cell contacts and epithelial cell state. This suggests a loss of adhesion in the general RPE monolayer, perhaps indicative of cells undergoing transition to a more migratory phenotype. Taken together, these findings might indicate that RPE-cells undergo EMT, leading to their proliferation and accumulation along subretinal and retinal vessels. Once migrating RPE-cells have found their new position, they may develop cell-cell contacts with neighboring RPE-cells, forming dense plaques around the vessels, and possibly transition back to an epithelial state (“mesenchymal-epithelial transition”, MET). Similar mechanisms have been previously described to occur during cancer metastasis and organ morphogenesis [33, 34]. The hypothesis that RPE cells undergo different stages of EMT, followed by MET, in the *Vldlr*^−/−^ model is further supported by the differences we found in the expression of epithelial and mesenchymal markers in *Vldlr*^−/−^ mice compared to previous reports in MacTel. In MacTel, intraretinal pigment plaques were found to only express the epithelial marker CK18, while lacking any mesenchymal markers [2]. In the *Vldlr*^−/−^ model, on the other hand, we observed that single intraretinal pigmented lesions also expressed mesenchymal markers, possibly indicating the presence of different stages of lesions and different stages of EMT. This was particularly the case for lesions that were less dense, and thus possibly less mature, indicating that some RPE-cells were still in a mesenchymal state, before undergoing MET and forming dense, mature plaques around the vessels. In MacTel, on the other hand, intraretinal pigment plaques usually develop within months and may form and progress over the course of several years, forming dense, organized clusters of pigment around vessels [2, 7]. Thus, it is conceivable that lesions, once clinically detectable, are overall more mature, and RPE-cells within dense plaques no longer possess mesenchymal properties, possibly having transitioned back to an epithelial state. Future studies, including inhibition of EMT (and MET) at specific stages and at specific timepoints, may provide further insight and are warranted to further explore the proposed mechanisms.

EMT of RPE-cells has been described in different ocular conditions [5] and various inducers of EMT have previously been discussed [5, 25, 29, 30]. In the RPE of *Vldlr*^−/−^ mice, we found several known drivers of EMT to be enriched, with FGF2 and TNFA being the most significant ones in our model. While these factors could be related to general neovascularization events in the *Vldlr*^−/−^ retina, it is important to note that their inhibition actually increased neovascularization and leakage, thus indicating a role in EMT rather than general neovascularization. Other known drivers of EMT and/or fibrosis have previously been identified in the *Vldlr*^−/−^ model [35, 36]. Notably, EMT and TNFA/ NFκB pathways were previously found to be among the most significantly enriched gene sets in RPE cells differentiated from induced pluripotent stem cells from MacTel donors [37]. This underlines the clinical relevance and applicability of our findings in the *Vldlr*^−/−^ model.

Inhibiting EMT in the *Vldlr*^−/−^ model resulted in increased neovascular growth and exudation, confirming the beneficial effect of perivascular pigment accumulation we had clinically observed in patients with MacTel. Pigment plaques, on the other hand, have been shown to be associated with a focal loss in retinal sensitivity (“absolute scotomas” on fundus-controlled perimetry) [6] and the pigment area with a decrease in visual acuity in patients with MacTel [7], thus representing a mixed blessing.

Intravitreal VEGF inhibitors have been successfully used to treat numerous retinal diseases characterized by vascular involvement. In MacTel, VEGF inhibitors have been effectively applied to eyes showing subretinal neovascularization [38–40], while no functional improvement has been observed in non-neovascular eyes [41]. The latter showed, however, a (temporary) decrease in fluorescein leakage during treatment, followed by an increase in leakage once treatment had been discontinued [41]. Notably, treated eyes developed excessive pigmentation and fibrosis later on [41, 42]. In the *Vldlr*^−/−^ model, we found that perivascular pigment only developed when vascular proliferation was present and pigment plaques only developed along proliferating vessels. Taken together, this might further indicate that interfering with the proposed natural “repair mechanism” of the diseased retina/RPE, by suppressing either EMT or vascular leakage/ intraretinal proliferation, may have detrimental effects and thus should be avoided in MacTel and related diseases.

## Conclusions

In summary, we showed that perivascular pigment accumulation is associated with a decrease in vascular leakage and stabilization of neovascular growth in proliferative retinal diseases, such as MacTel, and respective animal models. We revealed underlying mechanisms leading to perivascular pigment accumulation, and discussed beneficial effects, changing our current understanding of these changes. Knowledge about pathophysiological mechanisms is crucial for understanding the disease course and developing therapeutic interventions as well as choosing appropriate timepoints for treatment. We conclude that interfering with this “natural repair mechanism” of the diseased RPE may have detrimental effects on the course of the disease and should thus be avoided.

## Materials and methods

### Participants

In a retrospective, longitudinal approach, imaging data of affected participants from twelve participating sites of the multi-center Natural History Study of Macular Telangiectasia (MacTel Study) were evaluated. Protocol details of this study have been published previously [43]. The diagnosis of MacTel type 2 was based on characteristic morphologic findings on fundoscopy, OCT, fundus autofluorescence and fluorescein angiography [6], and was confirmed by the Moorfields Eye Hospital Reading Centre, London, UK. Patients underwent annual study visits, and eyes were reviewed over a minimum observational period of 24 months.

Data were collected at a minimum of two time points (at baseline and last available follow up visits), and sites were selected based on the availability of longitudinal imaging data, including color fundus photography (CFP), Spectral Domain-OCT (SD-OCT; volume scans of 15° x 10° (high resolution mode, 97 scans) or 25° x 30° (high speed mode, 61 scans); Spectralis, Heidelberg Engineering, Heidelberg, Germany), fundus autofluorescence (FA) and fundus fluorescein angiography (FFA; 30°, centered on the fovea, images taken at 30 sec, 1, 5 and 10 min after fluorescein injection). In a small subset of participants, OCT-angiography (OCT-A) data were additionally available.

Inclusion criteria for this analysis were a confirmed diagnosis of MacTel, a full data set including the examinations listed above and sufficient image quality. As pigmentary changes have been shown to only develop in eyes showing disruptions of outer retinal layers [21], and to ensure equal baseline conditions among both groups, only eyes with intermediate disease stages were included. The latter was defined as visible disruption of the ellipsoid zone (EZ)/ photoreceptor layer on OCT, and disease stages 2–3 according to Gass and Blodi [12]. Eyes showing pigment plaques and/ or subretinal/ sub-RPE neovascular membranes (stages 4 and 5 according to Gass and Blodi) at baseline were excluded. Further exclusion criteria were the presence of potentially confounding retinal diseases including central serous chorioretinopathy, age-related macular degeneration or diabetic retinopathy, and previous therapies including VEGF-inhibitors, vitreo-retinal surgery, photodynamic therapy or central laser treatment.

Eyes were retrospectively divided into two groups, one group showing a de novo formation of pigment plaques at last follow up, and one group without any pigmentary changes, and outcome parameters were compared between both groups.

Outcome parameters included changes in leakage on FFA (decreased, increased, or stable leakage within subfields 1–5 of the ETDRS grid) and a de novo development of exudative subretinal neovascular membranes as observed on CFP, OCT, FFA, and OCT-A (if available).

### Image grading and definitions

#### Pigment plaques

CFP and FA images were analyzed for the presence and position of pigment plaques at baseline and last follow up.

#### Leakage on FFA

On FFA, vessels were graded for an (1) increase, (2) decrease, or (3) no visible changes in fluorescein leakage at last follow up compared to baseline. Images were evaluated and compared at different time points (at 30 sec, 1 min, and 10 min after fluorescein injection) and within subfields 1–5 of the ETDRS grid.

#### Neovascularization and exudation

Previously described criteria were applied to identify neovascular membranes on OCT, FFA, CFP, and OCT-A images (if available) [15, 21]. Neovascular exudation was defined as focal retinal thickening, sub- or intraretinal fluid, and/or hemorrhages as observed on OCT and/ or CFP in neovascular eyes.

#### Animals

*Vldlr*^–/–^ mice and control littermates (*Vldlr*^−/+^ mice) (The Jackson Laboratory) of up to 12 months of age were used for all animal experiments.

#### Intravitreal injections

All intravitreal injections were performed using a Hamilton syringe and a 34-gauge needle (Hamilton), injecting 1 µl of solutions containing neutralizing antibodies against TNFa (250ng; MAB4101; R&D systems), FGF2 (200ng; clone bFM-1; 05-117, Millipore Sigma) or VEGF164 (200ng; AF-493-NA; R&D systems). For each of these antibodies, IgG isotype control antibodies were used as negative controls (MAB005R, R&D systems; 12–371, Millipore Sigma; AB-108-C, R&D systems).

Intravitreal injections with antibodies against FGF2 or TNFa were performed at P21, after subretinal neovascular tufts have formed, but before pigmented cells have started proliferating or migrating [17, 44]. Retinas and RPE/choroid/sclera complexes were analyzed for gene expression or immunofluorescence at P42. At this timepoint, RPE-cells have been shown to proliferate and accumulate along subretinal neovessels and have started migrating into the neuroretina, where they accumulate along retinal vessels [17–19, 44]. 

Intravitreal injections with antibodies against VEGF were performed at P12, when retinal vessels have started proliferating and anti-VEGF treatment has been shown to be most effective in this model [19, 35]. Retinas were evaluated at P28 using immunofluorescence imaging. At this timepoint, the normal RPE monolayer is disrupted, and clumps of RPE cells accumulate along neovascular tufts in the subretinal space of *Vldlr*^−/−^ mice [18, 19]. 

#### Dextran angiography

For analyzing vascular leakage, Fluorescein isothiocyanate–dextran (FITC; 40,000 MW; Sigma-Aldrich) was perfused through the left ventricle of deeply anesthetized *Vldlr*^−/−^ animals using 150 µl of a 50 mg/ml solution in PBS.

#### Immunofluorescence

Retinas and RPE/choroid complexes were dissected and prepared for whole mounts or sectioning. For preparation of retinal cross-sections, isolated eyes were fixed in 4% paraformaldehyde (PFA) for 4 h, placed in 15% sucrose overnight at 4 °C, followed by 30% sucrose for 2 h, and embedded in Tissue-Tek OCT compound (Sakura Finetek) for subsequent cryosectioning. For preparation of whole mounts, eyes were fixed in 4% PFA for one hour, and retina/RPE/choroid complexes were dissected and laid flat. Whole-mount retina/RPE/choroid complexes or cryosections were incubated in blocking buffer (10% fetal bovine serum and 0.1% Triton X-100 in phosphate-buffered saline [PBS]) at 4 °C for 2 h, followed by an incubation with primary antibodies in blocking buffer at 4 °C overnight. Specimens were then washed with PBS and incubated with Alexa Fluor–conjugated secondary antibodies (Thermo Fisher) for 2 h. Nuclei were stained using DAPI (Vector Laboratories). Specimen were mounted in Vectashield Plus Antifade Mounting Medium (Vectorlabs). Primary antibodies against ZO-1 (40-2200, Thermo Fisher), RPE-65 (PA5-110315, Thermo Fisher), alpha-smooth muscle actin (ab124964, Abcam), IBA1 (MA5-27726, Thermo Fisher) and Cytokeratin 18 (10830-1-AP, Proteintech) were used. Endothelial cells were labeled using Fluorescent-conjugated isolectin Griffonia Simplicifolia IB-4 (GS-Lectin) (I21412, Thermo Fisher). Images were captured with a confocal laser scanning microscope (LSM 710, Zeiss) and processed with the ZEN 2010 software (Zeiss). The NIH ImageJ software was used for quantifying dextran-leakage (number of pixels positive for dextran), pigment accumulation (number of pixels positive for pigment) and neovascular tufts (total numbers of visible tufts per eye).

### Gene expression analyses

For quantitative polymerase chain reaction (qPCR), retinas and RPE/choroid complexes were isolated in 500 µl Trizol. RNA isolation was performed using a RNeasy Plus Micro Kit (Qiagen) following the manufacturer’s instructions. 400ng RNA was used for real time-qPCR using the High-capacity cDNA Reverse transcriptase kit (Thermo Fisher). SYBR Green–based (Thermo Fisher) real-time quantitative PCR was performed on a QuantStudio 5 (Thermo Fisher) to analyze mRNA expression levels of various gene products. Expression levels were normalized to Cyclophilin A. Sequences of primers used are listed in Online Resource 4. Data were analyzed using the QuantStudio Design and Analysis Software 2.6.0 (Thermo Fisher).

### Statistics

Statistical analyses were performed using GraphPad Prism, version 10.1.0 (GraphPad Software, San Diego, CA, USA). Continuous variables were described by using the mean ± standard deviation (SD), median and interquartile range [IQR] and/ or ranges. Categorical variables were described in terms of frequency. Unpaired t-test with Welch’s correction was applied for comparing parameters between two groups in normally distributed samples. Mann–Whitney test was used to compare parameters between two groups in non-normally distributed samples. Kruskal-Wallis test with Dunn’s correction for multiple comparisons was used to compare data between multiple groups. Fisher’s exact test was applied to analyze contingency tables with small sample sizes. Pearson correlation coefficient was calculated to determine linear correlation between two parameters. Statistical tests applied for each experiment are detailed in the figure and table legends. A p-value < 0.05 was accepted as statistically significant.

## Electronic supplementary material

Below is the link to the electronic supplementary material.

Supplementary Material

## Data Availability

No datasets were generated or analysed during the current study.

## References

[1] George SM, Lu F, Rao M, Leach LL, Gross JM (2021) The retinal pigment epithelium: development, injury responses, and regenerative potential in mammalian and non-mammalian systems. Prog Retin Eye Res 85:100969. 10.1016/j.preteyeres.2021.100969 33901682 10.1016/j.preteyeres.2021.100969 PMC8536801

[2] Yasvoina M, Yang Q, Woods SM, Heeren T, Comer GM, C AE, Fruttiger M (2023) Intraretinal pigmented cells in retinal degenerative disease. Br J Ophthalmol 107(11):1736–1743. 10.1136/bjophthalmol-2021-320392 35301216 10.1136/bjophthalmol-2021-320392

[3] Augustin M, Fialova S, Himmel T, Glosmann M, Lengheimer T, Harper DJ, Plasenzotti R, Pircher M, Hitzenberger CK, Baumann B (2016) Multi-functional OCT enables longitudinal study of retinal changes in a VLDLR knockout mouse model. PLoS ONE 11(10):e0164419. 10.1371/journal.pone.0164419 27711217 10.1371/journal.pone.0164419 PMC5053493

[4] Tamiya S, Liu L, Kaplan HJ (2010) Epithelial-mesenchymal transition and proliferation of retinal pigment epithelial cells initiated upon loss of cell-cell contact. Invest Ophthalmol Vis Sci 51(5):2755–2763. 10.1167/iovs.09-4725 20042656 10.1167/iovs.09-4725

[5] Zhou M, Geathers JS, Grillo SL, Weber SR, Wang W, Zhao Y, Sundstrom JM (2020) Role of epithelial-mesenchymal transition in Retinal Pigment Epithelium Dysfunction. Front Cell Dev Biol 8:501. 10.3389/fcell.2020.00501 32671066 10.3389/fcell.2020.00501 PMC7329994

[6] Charbel Issa P, Gillies MC, Chew EY, Bird AC, Heeren TF, Peto T, Holz FG, Scholl HP (2013) Macular telangiectasia type 2. Prog Retin Eye Res 34:49–77. 10.1016/j.preteyeres.2012.11.002 23219692 10.1016/j.preteyeres.2012.11.002 PMC3638089

[7] Leung I, Sallo FB, Bonelli R, Clemons TE, Pauleikhoff D, Chew EY, Bird AC, Peto T, MacTel Study G (2018) Characteristics of pigmented lesions in type 2 idiopathic Macular Telangiectasia. Retina 38(Suppl 1):S43–S50. 10.1097/IAE.0000000000001842 29095354 10.1097/IAE.0000000000001842 PMC5726940

[8] Tzaridis S, Wintergerst MWM, Mai C, Heeren TFC, Holz FG, Charbel Issa P, Herrmann P (2019) Quantification of retinal and Choriocapillaris Perfusion in different stages of Macular Telangiectasia Type 2. Invest Ophthalmol Vis Sci 60(10):3556–3562. 10.1167/iovs.19-27055 31415079 10.1167/iovs.19-27055

[9] Breazzano MP, Yannuzzi LA, Spaide RF (2020) Characterizing retinal-choroidal anastomosis in Macular Telangiectasia Type 2 with Optical Coherence Tomography Angiography. Retina 40(1):92–98. 10.1097/IAE.0000000000002619 31246676 10.1097/IAE.0000000000002619

[10] Breazzano MP, Yannuzzi LA, Spaide RF (2020) Genesis of Retinal-Choroidal Anastomosis in Macular Telangiectasia Type 2: a longitudinal analysis. Retina. 10.1097/IAE.0000000000002986 32976212 10.1097/IAE.0000000000002986

[11] Tzaridis S, Heeren T, Mai C, Thiele S, Holz FG, Charbel Issa P, Herrmann P (2021) Right-angled vessels in macular telangiectasia type 2. Br J Ophthalmol 105(9):1289–1296. 10.1136/bjophthalmol-2018-313364 30808615 10.1136/bjophthalmol-2018-313364 PMC8380913

[12] Gass JD, Blodi BA (1993) Idiopathic juxtafoveolar retinal telangiectasis. Update of classification and follow-up study. Ophthalmology 100(10):1536–1546 8414413

[13] Engelbrecht NE, Aaberg TM Jr., Sung J, Lewis ML (2002) Neovascular membranes associated with idiopathic juxtafoveolar telangiectasis. Arch Ophthalmol 120(3):320–324 11879135 10.1001/archopht.120.3.320

[14] Davidorf FH, Pressman MD, Chambers RB (2004) Juxtafoveal telangiectasis-a name change? Retina 24(3):474–478 15187680 10.1097/00006982-200406000-00028

[15] Heeren TFC, Chew EY, Clemons T, Fruttiger M, Balaskas K, Schwartz R, Egan CA, Charbel Issa P, MacTel Study G (2020) Macular Telangiectasia Type 2: Visual Acuity, Disease End Stage, and the MacTel Area: MacTel Project Report Number 8. Ophthalmology. 10.1016/j.ophtha.2020.03.040 10.1016/j.ophtha.2020.03.040 PMC8380038 32586743

[16] Mueller S, Gunnemann F, Rothaus K, Book M, Faatz H, Bird A, Pauleikhoff D (2021) Incidence and phenotypical variation of outer retina-associated hyperreflectivity in macular telangiectasia type 2. Br J Ophthalmol 105(4):573–576. 10.1136/bjophthalmol-2020-317997 33414243 10.1136/bjophthalmol-2020-317997

[17] Heckenlively JR, Hawes NL, Friedlander M, Nusinowitz S, Hurd R, Davisson M, Chang B (2003) Mouse model of subretinal neovascularization with choroidal anastomosis. Retina 23(4):518–522. 10.1097/00006982-200308000-00012 12972764 10.1097/00006982-200308000-00012

[18] Hu W, Jiang A, Liang J, Meng H, Chang B, Gao H, Qiao X (2008) Expression of VLDLR in the retina and evolution of subretinal neovascularization in the knockout mouse model’s retinal angiomatous proliferation. Invest Ophthalmol Vis Sci 49(1):407–415. 10.1167/iovs.07-0870 18172119 10.1167/iovs.07-0870

[19] Dorrell MI, Aguilar E, Jacobson R, Yanes O, Gariano R, Heckenlively J, Banin E, Ramirez GA, Gasmi M, Bird A, Siuzdak G, Friedlander M (2009) Antioxidant or neurotrophic factor treatment preserves function in a mouse model of neovascularization-associated oxidative stress. J Clin Invest 119(3):611–623. 10.1172/JCI35977 19188685 10.1172/JCI35977 PMC2648679

[20] Krivosic V, Lavia C, Aubineau A, Tadayoni R, Gaudric A (2021) OCT of outer retinal hyperreflectivity, Neovascularization, and pigment in Macular Telangiectasia Type 2. Ophthalmol Retina 5(6):562–570. 10.1016/j.oret.2020.09.012 32956858 10.1016/j.oret.2020.09.012

[21] Tzaridis S, Hess K, Heeren TFC, Bonelli R, Holz FG, Friedlander M (2021) Hyperreflectivity on Optical Coherence Tomography in Macular Telangiectasia Type 2. Retina 41(7):1428–1437. 10.1097/IAE.0000000000003111 33438900 10.1097/IAE.0000000000003111

[22] Sun Y, Lin Z, Liu CH, Gong Y, Liegl R, Fredrick TW, Meng SS, Burnim SB, Wang Z, Akula JD, Pu WT, Chen J, Smith LEH (2017) Inflammatory signals from photoreceptor modulate pathological retinal angiogenesis via c-Fos. J Exp Med 214(6):1753–1767. 10.1084/jem.20161645 28465464 10.1084/jem.20161645 PMC5461000

[23] Li C, Huang Z, Kingsley R, Zhou X, Li F, Parke DW, Cao II W (2007) Biochemical alterations in the retinas of very low-density lipoprotein receptor knockout mice: an animal model of Retinal Angiomatous Proliferation. Arch Ophthalmol 125(6):795–803. 10.1001/archopht.125.6.795 17562991 10.1001/archopht.125.6.795

[24] Chen HC, Zhu YT, Chen SY, Tseng SC (2012) Wnt signaling induces epithelial-mesenchymal transition with proliferation in ARPE-19 cells upon loss of contact inhibition. Lab Invest 92(5):676–687. 10.1038/labinvest.2011.201 22391957 10.1038/labinvest.2011.201 PMC3961713

[25] Matsuda Y, Nonaka Y, Futakawa S, Imai H, Akita K, Nishihata T, Fujiwara M, Ali Y, Bhisitkul RB, Nakamura Y (2019) Anti-angiogenic and Anti-scarring Dual Action of an anti-fibroblast growth factor 2 Aptamer in Animal models of Retinal Disease. Mol Ther Nucleic Acids 17:819–828. 10.1016/j.omtn.2019.07.018 31454678 10.1016/j.omtn.2019.07.018 PMC6716068

[26] Schultz GS, Grant MB (1991) Neovascular growth factors. Eye (Lond) 5(Pt 2):170–180. 10.1038/eye.1991.31 1712736 10.1038/eye.1991.31

[27] Wang CH, Cao GF, Jiang Q, Yao J (2012) TNF-alpha promotes human retinal pigment epithelial (RPE) cell migration by inducing matrix metallopeptidase 9 (MMP-9) expression through activation of Akt/mTORC1 signaling. Biochem Biophys Res Commun 425(1):33–38. 10.1016/j.bbrc.2012.07.044 22820188 10.1016/j.bbrc.2012.07.044

[28] Ni Y, Qin Y, Huang Z, Liu F, Zhang S, Zhang Z (2020) Distinct serum and vitreous inflammation-related factor profiles in patients with proliferative vitreoretinopathy. Adv Ther 37(5):2550–2559. 10.1007/s12325-020-01325-x 32274748 10.1007/s12325-020-01325-x PMC7467460

[29] Limb GA, Alam A, Earley O, Green W, Chignell AH, Dumonde DC (1994) Distribution of cytokine proteins within epiretinal membranes in proliferative vitreoretinopathy. Curr Eye Res 13(11):791–798. 10.3109/02713689409025133 7851114 10.3109/02713689409025133

[30] Boles NC, Fernandes M, Swigut T, Srinivasan R, Schiff L, Rada-Iglesias A, Wang Q, Saini JS, Kiehl T, Stern JH, Wysocka J, Blenkinsop TA, Temple S (2020) Epigenomic and transcriptomic changes during human RPE EMT in a stem cell model of Epiretinal Membrane Pathogenesis and Prevention by Nicotinamide. Stem Cell Rep 14(4):631–647. 10.1016/j.stemcr.2020.03.009 10.1016/j.stemcr.2020.03.009 PMC7160390 32243845

[31] Meleth AD, Toy BC, Nigam D, Agron E, Murphy RP, Chew EY, Wong WT (2013) Prevalence and progression of pigment clumping associated with idiopathic macular telangiectasia type 2. Retina 33(4):762–770. 10.1097/IAE.0b013e3182695bb3 23064429 10.1097/IAE.0b013e3182695bb3 PMC3549320

[32] Jaissle GB, May CA, van de Pavert SA, Wenzel A, Claes-May E, Giessl A, Szurman P, Wolfrum U, Wijnholds J, Fischer MD, Humphries P, Seeliger MW (2010) Bone spicule pigment formation in retinitis pigmentosa: insights from a mouse model. Graefes Arch Clin Exp Ophthalmol 248(8):1063–1070. 10.1007/s00417-009-1253-9 20012642 10.1007/s00417-009-1253-9

[33] Pei D, Shu X, Gassama-Diagne A, Thiery JP (2019) Mesenchymal-epithelial transition in development and reprogramming. Nat Cell Biol 21(1):44–53. 10.1038/s41556-018-0195-z 30602762 10.1038/s41556-018-0195-z

[34] Yao D, Dai C, Peng S (2011) Mechanism of the mesenchymal-epithelial transition and its relationship with metastatic tumor formation. Mol Cancer Res 9(12):1608–1620. 10.1158/1541-7786.MCR-10-0568 21840933 10.1158/1541-7786.MCR-10-0568

[35] Usui-Ouchi A, Usui Y, Kurihara T, Aguilar E, Dorrell MI, Ideguchi Y, Sakimoto S, Bravo S, Friedlander M (2020) Retinal microglia are critical for subretinal neovascular formation. JCI Insight 5(12). 10.1172/jci.insight.137317 10.1172/jci.insight.137317 PMC7406258 32437334

[36] Chen Q, Jiang N, Zhang Y, Ye S, Liang X, Wang X, Lin X, Zong R, Chen H, Liu Z (2020) Fenofibrate inhibits Subretinal Fibrosis through suppressing TGF-beta-Smad2/3 signaling and wnt signaling in Neovascular Age-Related Macular Degeneration. Front Pharmacol 11:580884. 10.3389/fphar.2020.580884 33442383 10.3389/fphar.2020.580884 PMC7797782

[37] Eade KT, Ansell BRE, Giles S, Fallon R, Harkins-Perry S, Nagasaki T, Tzaridis S, Wallace M, Mills EA, Farashi S, Johnson A, Sauer L, Hart B, Diaz-Rubio ME, Bahlo M, Metallo C, Allikmets R, Gantner ML, Bernstein PS, Friedlander M (2023) iPSC-derived retinal pigmented epithelial cells from patients with macular telangiectasia show decreased mitochondrial function. J Clin Invest 133(9). 10.1172/JCI163771 10.1172/JCI163771 PMC10145939 37115691

[38] Barth T, Zeman F, Helbig H, Gamulescu MA (2018) Intravitreal anti-VEGF treatment for subretinal neovascularisation secondary to type 2 idiopathic juxtafoveolar telangiectasia. Int Ophthalmol 38(1):191–198. 10.1007/s10792-017-0447-0 10.1007/s10792-017-0447-0 28108904

[39] Narayanan R, Chhablani J, Sinha M, Dave V, Tyagi M, Pappuru RR, Kuppermann BD (2012) Efficacy of anti-vascular endothelial growth factor therapy in subretinal neovascularization secondary to macular telangiectasia type 2. Retina 32(10):2001–2005. 10.1097/IAE.0b013e3182625c1d 10.1097/IAE.0b013e3182625c1d 22990322

[40] Roller AB, Folk JC, Patel NM, Boldt HC, Russell SR, Abramoff MD, Mahajan VB (2011) Intravitreal bevacizumab for treatment of proliferative and nonproliferative type 2 idiopathic macular telangiectasia. Retina 31(9):1848–1855. 10.1097/IAE.0b013e31820d3feb 10.1097/IAE.0b013e31820d3feb 21610563

[41] Charbel Issa P, Finger RP, Kruse K, Baumuller S, Scholl HP, Holz FG (2011) Monthly ranibizumab for nonproliferative macular telangiectasia type 2: a 12-month prospective study. Am J Ophthalmol 151(5):876–886, e871. 10.1016/j.ajo.2010.11.019 10.1016/j.ajo.2010.11.019 21334595

[42] Kupitz EH, Heeren TF, Holz FG, Charbel Issa P (2015) Poor long-term outcome of anti-vascular endothelial growth factor therapy in nonproliferative macular telangiectasia type 2. Retina 35(12):2619–2626. 10.1097/IAE.0000000000000715 10.1097/IAE.0000000000000715 26340529

[43] Clemons TE, Gillies MC, Chew EY, Bird AC, Peto T, Figueroa MJ, Harrington MW, MacTel Research G (2010) Baseline characteristics of participants in the natural history study of macular telangiectasia (MacTel) MacTel Project Report 2. Ophthalmic Epidemiol 17(1):66–73. 10.3109/09286580903450361 20100102 10.3109/09286580903450361 PMC8329604

[44] Li C, Huang Z, Kingsley R, Zhou X, Li F, Parke DW 2nd, Cao W (2007) Biochemical alterations in the retinas of very low-density lipoprotein receptor knockout mice: an animal model of retinal angiomatous proliferation. Arch Ophthalmol 125(6):795–803. 10.1001/archopht.125.6.795 17562991 10.1001/archopht.125.6.795

