# Supplementary material for: Retinal pigment epithelial cells reduce vascular leak and proliferation in retinal neovessels
Source: Angiogenesis. 2024 Nov 27;28(1):1. doi: 10.1007/s10456-024-09954-4 (PMC11602807; doi:10.1007/s10456-024-09954-4)
Supplement: Supplementary file 1 — Supplementary Material [file 10456_2024_9954_MOESM1_ESM.pdf]

Article title: Retinal pigment epithelial cells reduce vascular leak and proliferation in retinal neovessels

Journal name: Angiogenesis

Author names: Simone Tzaridis*, Edith Aguilar, Michael I Dorrell, Martin Friedlander, Kevin T Eade

***Corresponding author:** Simone Tzaridis, The Lowy Medical Research Institute, La Jolla, CA, USA; email: stzaridis@scripps.edu

Supplemental material:

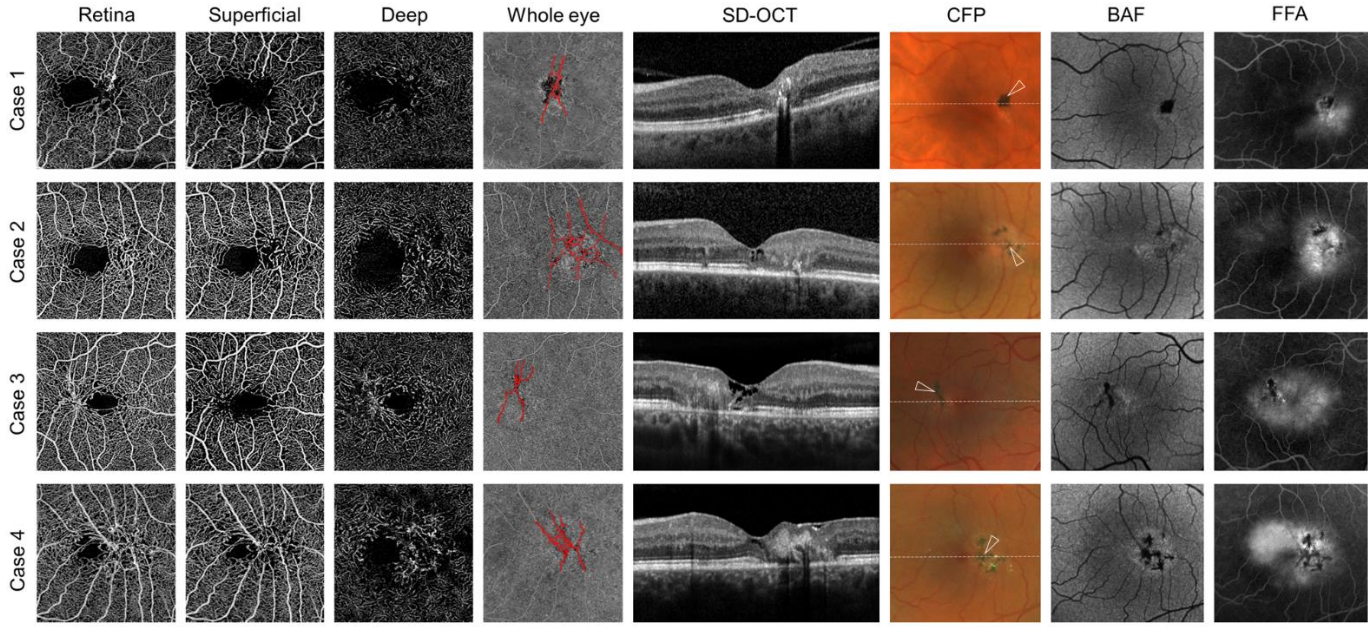

Online Resource 1: Pigment accumulates along proliferating retinal vessels and retinal-choroidal anastomoses in eyes with MacTel.

Multimodal retinal images of four exemplary eyes with MacTel, including optical coherence tomography-angiography (en face scans of the following layers are shown: whole retina, superficial retinal plexus, deep retinal plexus and whole eye scans; vessels forming retinal-retinal and retinal-choroidal anastomoses are marked in red), B-scan Spectral domain (SD-) OCT, color fundus photographs (CFP), blue-light autofluorescence (BAF), and fundus fluorescein angiograms (FFA, late phase). On CFP images, pigment plaques are indicated with white arrow heads, and horizontal lines indicate the position of each B-scan. On OCT, pigment plaques depict as hyper-reflective lesions.

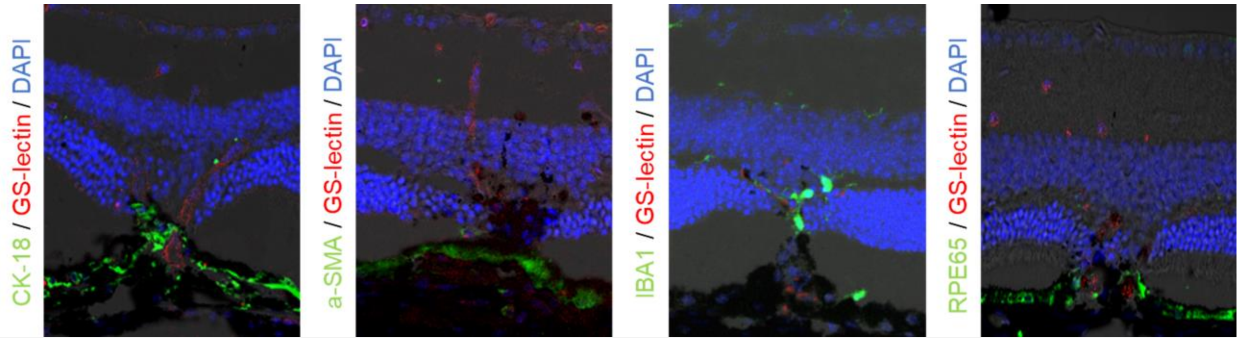

Online Resource 2: Dense intraretinal pigment plaques express the retinal pigment epithelium marker cytokeratin18 (CK18).

Immunofluorescence imaging of retinal sections of 11-months-old *Vldlr*$^{-/-}$ mice with GS-lectin (red) and CK18, alpha-smooth muscle actin (ASMA), IBA1 or RPE65 (green). Nuclei are shown in blue (DAPI). Intraretinal pigment co-locates with CK18, but not with ASMA, IBA1 or RPE65.

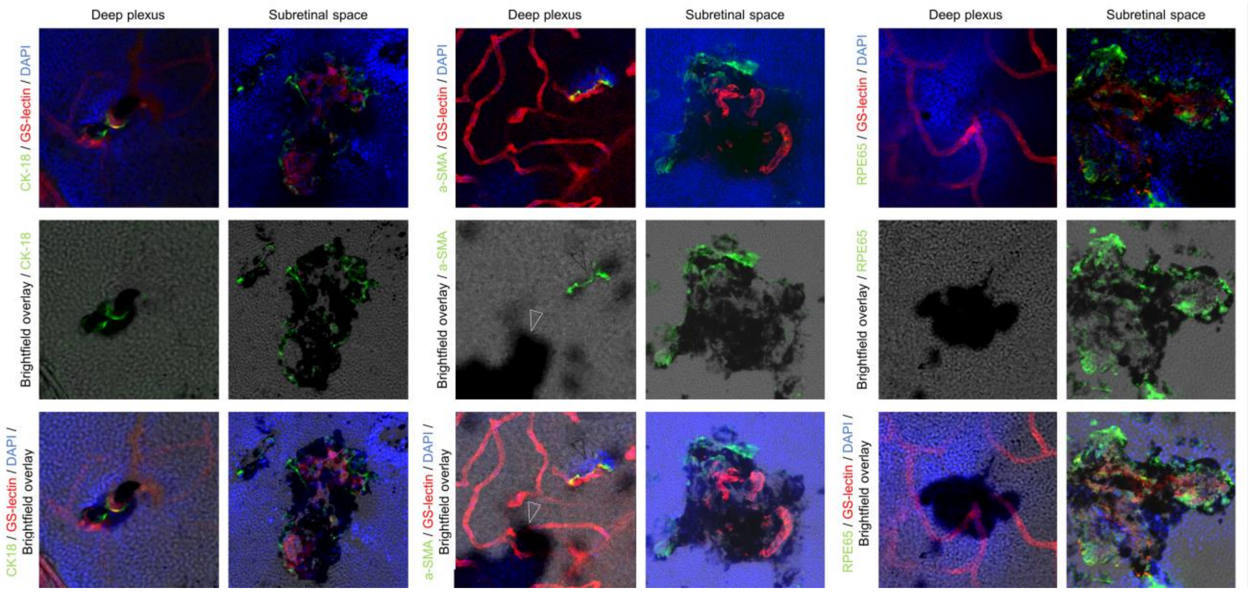

Online Resource 3: RPE-cells form clusters around neovessels within the subretinal space, and express epithelial and mesenchymal markers.

Dense intraretinal pigment plaques (along vessels of the deep plexus) express the RPE marker cytokeratin18 (CK18), but not RPE65. The expression of ASMA was observed in single pigmented intraretinal lesions (black arrowhead). The latter appeared, however, overall less dense compared to those lesions lacking ASMA expression (white arrowhead). Immunofluorescence imaging of flat mounted retinas of 12-months-old *Vldlr*$^{-/-}$ mice with GS-lectin (red) and CK18, alpha-smooth muscle actin (ASMA), IBA1 or RPE65 (green). Nuclei are shown in blue (DAPI).

Online Resource 4:

Sequence of primers used for RT-PCR.

Gene	Forward primer (5’-3’)	Reverse primer (5’-3’)
TNF-alpha	CCCTCACACTCAGATCATCTTCT	GCTACGACGTGGGCTACAG
FGF-2	CAGTTCGTTTCAGTGCCACA	GGCTGCTGGCTTCTAAGTGT
EGF	ATTTCGTTGTTAGCACCATCCC	GGCACAACCAGGCAAAGGAT
TGF-beta-2	TCCCCTCCGAAAATGCCATC	TGCTATCGATGTAGCGCTGG
N-cadherin	TCTCCAAGTGGCCAGGAAAC	CAAAGCTTCCGGGCGTAGA
ZO-1	GACCTCTGCAGCAATAAAGCA	AGAAATCGTGCTGATGTGCCA
E-cadherin	AACCCAAGCACGTATCAGGG	GAGTGTTGGGGGCATCATCA
vimentin	GGCTGCGAGAGAAATTGCAG	TTCAAGGTCAAGACGTGCCA
SMAD2	TTGCTGTTGTTGTTGTTTAAGGA	AGCAATACTGCCTCTTGTTGC
SMAD4	AGTAATCGCGCATCAACGGA	GAATACTGGCCGGCTGACTT
ZEB1	CTGAGCACAGACTACCGCAA	GGTCTGCTGGCAGTTCATCA
ZEB2	AAGCGTTTGCGGAGACTTCA	AACACGCGCCACCTATCTTT
SNAIL1	AGTTGACTACCGACCTTGCG	TGCAGCTCGCTATAGTTGGG
SNAIL2	AGAAGCCCAACTACAGCGAA	ATAGGGCTGTATGCTCCCGA
beta-catenin	CGCCGCTTATAAATCGCTCC	TTCACAGGACACGAGCTGAC
LEF1	CAGCGCGAGACAATTATGGC	TAGGCAGCTGTCATTCTGGG
fibronectin	CTGGATCCCCTCCCAGAGAA	TTGGGGTGTGGAAGGGTAAC
TGF-beta1	ACTGGAGTTGTACGGCAGTG	GGGGCTGATCCCGTTGATTT
PDGF-A	TTCGTCGATAACACGCACGA	TTCCCAGAGTCCCCTCATGT
PDGF-B	CAACGAGAAAGCCGGAGCAG	GTCTATCTACCCACTCGCTCG
PDGF-C	CCAGTCAGCCAAATGCTCCT	TGGGTATAGTTCCTCCCGTTCT
PDGF-D	TGAGAGCAATCACCTCACAGAC	CAGAAGCAGGTTCCTTGGGT
